# The Effect of Retinoids in Vascular Smooth Muscle Cells: From Phenotyping Switching to Proliferation and Migration

**DOI:** 10.3390/ijms251910303

**Published:** 2024-09-25

**Authors:** Ioanna Samara, Amalia I. Moula, Anargyros N. Moulas, Christos S. Katsouras

**Affiliations:** 1 Faculty of Medicine, School of Health Sciences, University of Ioannina, 45110 Ioannina, Greece; ioan.samara31@gmail.com; 2 Department of Surgery, “Achillopouleion” General Hospital, 38222 Volos, Greece; amaliamoula1@gmail.com; 3 General Department, University of Thessaly, 41500 Larissa, Greece; moulas@uth.gr

**Keywords:** atherosclerosis, retinoic acid, vascular smooth muscle cells

## Abstract

Atherosclerosis, a term derived from the Greek “athero” (atheroma) and “sclerosis” (hardening), is a long-standing process that leads to the formation of atheromatous plaques in the arterial wall, contributing to the development of atherosclerotic cardiovascular disease. The proliferation and migration of vascular smooth muscle cells (VSMCs) and the switching of their phenotype play a crucial role in the whole process. Retinoic acid (RA), a natural derivative of vitamin A, has been used in the treatment of various inflammatory diseases and cell proliferation disorders. Numerous studies have demonstrated that RA has an important inhibitory effect on the proliferation, migration, and dedifferentiation of vascular smooth muscle cells, leading to a significant reduction in atherosclerotic lesions. In this review article, we explore the effects of RA on the pathogenesis of atherosclerosis, focusing on its regulatory action in VSMCs and its role in the phenotypic switching, proliferation, and migration of VSMCs. Despite the potential impact that RA may have on the process of atherosclerosis, further studies are required to examine its safety and efficacy in clinical practice.

## 1. Introduction

Atherosclerosis, a complex disease that involves both inflammatory and non-inflammatory mechanisms, remains the leading cause of death worldwide [1,2,3]. Development of atherosclerotic plaques is characterized by the accumulation of monocytes, which later differentiate into macrophages, T-cells, and mast cells; the infiltration of lipids in the arterial wall; and a fibrous cap formed by vascular smooth muscle cells (VSMCs) and collagen [4]. In recent decades, many studies have proven the ability of VSMCs to differentiate into various phenotypes, proliferate, and migrate in response to various stimuli, participating in all stages of atherosclerosis [5,6]. More than 50% of foam cells originate from the cells of the intimal SMCs and from endothelial cells [7]. Moreover, the activation of VSMCs in response to endothelial injury after angioplasty is a major contributor to restenosis [8].

Retinoic acid (RA) promotes the signaling of specific nuclear receptors, which influence gene expression capable of regulating cell proliferation and differentiation [9,10], and it has been used in clinical practice as an anticancer agent [11,12]. Experimental studies have shown that RA can significantly inhibit atherosclerotic lesions [13,14,15]. In addition, RA inhibits both the proliferation and migration of VSMCs, as shown in in vitro cultures of human arterial smooth muscle cells, and regulates their differentiation [16]. Recently, local delivery of RA through a drug-eluting stent has been used in a rabbit iliac artery model [17].

In this review article, we discuss the role of RA in the phenotypic switching, proliferation, and migration of VSMCs.

## 2. Methods

We conducted a literature search across two electronic databases: PubMed and Cochrane Library. No date range was set. We used various combinations of the following keywords to analyze relevant articles: “retinoic acid” or “vitamin A” or “retinoid” or “ATRA” or “RA” or “RAR” and “smooth muscle cell” or “smooth muscle” or “klf4” or “cardiac” or “cardiovascular” or “restenosis” or “stenosis” or “atherosclerosis” or “proliferation” or “migration” or “aortic” or femoral” or “coronary” or “experimental.” Additionally, reference lists of retrieved review articles were evaluated to identify potential additional studies. We excluded non-English language publications.

## 3. Chemistry and Metabolism of Retinoids

**Figure 1:**
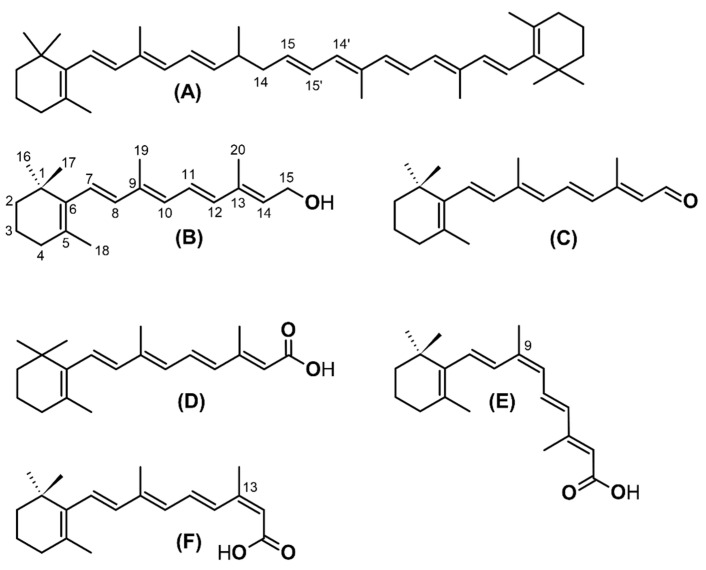
Chemical structure of biologically important carotenoids and retinoids. (**A**): β-carotene, (**B**) all-trans-retinol, (**C**) all-trans-retinaldehyde (retinal), (**D**) all-trans-retinoic acid, (**E**) 9-cis retinoic acid, and (**F**) 13-cis retinoic acid.

Retinoids are a class of natural and synthetic compounds chemically related to all-trans-retinol (vitamin A). Their molecules consist of four isoprenoid units joined in a head-to-tail manner and can be formally derived from a monocyclic parent compound containing five carbon–carbon double bonds and a functional group at the terminus of the acyclic portion. Common retinoids with biological significance are retinol, retinaldehyde (or retinal), retinoic acid, and retinyl esters. As a result of the presence of multiple double bonds, retinol and its derivatives can exist in different geometric isomer forms. The major naturally occurring isomers are the all-trans-ones, while other isomers are present in lower concentrations but have distinct physiological roles (Figure 1) [18,19].

Vitamin A is a nutrient essential for important physiological functions, including vision, reproduction, immune function, growth, and development. The human body cannot synthesize retinoids, and they need to be taken from food either as preformed retinoids or in the form of molecules with provitamin A activity, particularly carotenoids. As an overview, vitamin A is absorbed in the intestine and, through the lymphatic system, passes to the general circulation in lipoproteins and reaches the target organs, where it exerts its biological actions. Excess of vitamin A is stored mainly in the liver cells but also in other cells, such as the fat cells.

Several foods of plant origin contain carotenoids, pigments that are responsible for the characteristic yellow, orange, red, and purple color of plants, fruits, and flowers. Some carotenoids are provitamin A and can be converted to retinoids. A major nutritional source of retinoids is the orange plant carotenoid β-carotene. In the intestinal epithelial cells, β-carotene is converted to retinaldehyde (also known as retinal) by beta-carotene 15,15’-monooxygenase 1 (BCMO1) with the cleavage of the central carbon double bond. Retinaldehyde can then either be oxidized to retinoic acid by retinaldehyde dehydrogenase enzymes or be reduced by retinaldehyde reductases to give retinol and, finally, retinyl esters. These esters then follow the metabolic fate of the preformed retinoids that are absorbed by food, as described below. Any non-converted β-carotene is released into the lymphatic system from the basolateral side of the enterocytes and, via the mesenteric lymph, enters the general circulation in the form of chylomicron lipoproteins.

Foods of animal origin, such as liver, meat, fish, eggs, dairy products, and fortified foods such as cereals, are sources of retinol and retinyl esters. In the intestine, retinyl esters undergo hydrolysis of retinol and fatty acids before they can be absorbed by the apical membrane of the enterocytes. The main enzymes involved in this hydrolysis are retinyl ester hydrolases (REHs). Retinol in the enterocytes can be oxidized to all-trans-retinoic acid but predominantly binds to cellular retinol-binding protein-2 (CRBP2) and is re-esterified mainly by lecithin–retinol acyltransferase (LRAT) and partially by other enzymes. The newly formed retinyl esters exit the enterocytes from the basolateral membrane and enter the circulation via the mesenteric lymph in the form of chylomicrons [20,21,22].

The chylomicrons containing the retinyl esters and carotenoids are secreted into the lymphatic system, and finally, they enter the general circulation and are delivered to the liver or to the target organs and their respective cells. The liver is the major site for retinol metabolism and storage. The hepatocytes uptake the chylomicrons containing retinyl esters and hydrolyze the esters to retinol, which is bound to CRBP1. Subsequently, retinol can be either released in the circulation bound to retinol binding protein 4 (RBP4) and transthyretin (TTR) or transferred to hepatic stellate cells and stored in the form of retinyl esters. The human body does not possess the ability to synthesize vitamin A, but unlike other vitamins, vitamin A can be stored. The liver stores more than two-thirds of the total vitamin A, mainly in the form of retinyl palmitate [20,22,23].

The catabolism of retinoids begins with cytochrome P450 cyp26-catalyzed hydroxylation of all-trans-retinoic acid (ATRA) and oxidation to 4-oxo-retinoic acid. Further oxidation produces shorter-chain degradation products that are excreted via the biliary or the renal tract [23].

## 4. Mode of Action of Retinoids

Vitamins have genomic and non-genomic effects on various tissues [24]. The active form of vitamin A is retinoic acid (RA). RA is the natural endogenous agonist of the retinoid nuclear receptors and acts by inducing transcription and activating or suppressing the expression of various genes. There are two families of retinoid nuclear receptors: the retinoic acid receptors (RARs) and the retinoid X receptors (RXRs). Each family has three isotypes, namely a, b, and c. All-trans-retinoic acid binds only to RARs, while 9-cis-retinoic acid binds to both RARs and RXRs. The RXRs can form dimers with other receptors, such as the thyroid hormone and vitamin D receptors, and indirectly affect the action of these receptors [22,25].

**Figure 2:**
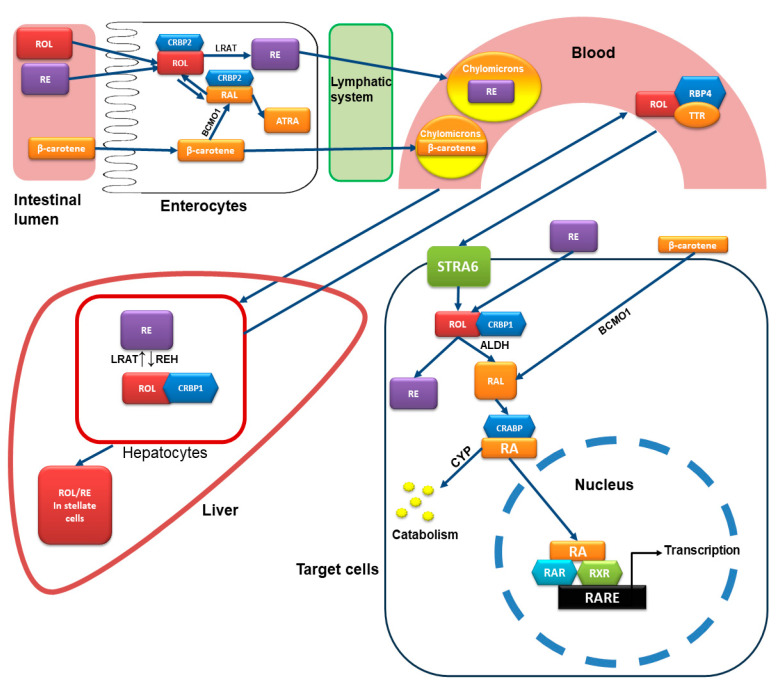
Metabolism and mode of action of retinoids. In the intestine, retinyl esters (RE) undergo hydrolysis to retinol (ROL) and fatty acids before they can be absorbed by the enterocytes. Retinol in the enterocytes can be oxidized to all-trans-retinoic acid (RA) but predominantly binds to cellular retinol-binding protein-2 (CRBP2) and is re-esterified mainly by lecithin–retinol acyltransferase (LRAT). The retinyl esters enter the circulation via the mesenteric lymph in the form of chylomicrons. In the intestinal epithelial cells, β-carotene is converted to retinaldehyde (RAL) by beta-carotene 15,15’-monooxygenase 1 (BCMO1). Retinaldehyde can then either be oxidized to retinoic acid (RA) or reduced to give retinol and, finally, retinyl esters. The chylomicrons containing the retinyl esters and carotenoids are secreted into the general circulation via the lymphatic system and are delivered to the liver or to the target organs and their respective cells. The hepatocytes uptake the chylomicrons containing retinyl esters and, using retinly ester hydrolases (REH), hydrolyze the esters to retinol, which is bound to CRBP1. Subsequently, retinol can be either released in the circulation bound to RBP4 and transthyretin (TTR) or transferred to hepatic stellate cells and stored in the form of retinyl esters. The target cells uptake RBP4-bound all-trans-retinol with the vitamin A receptor, stimulated by retinoic acid 6 (STRA6). Subsequently, retinol is bound to CRBP1 and undergoes oxidation in two steps: first to retinaldehyde by aldehyde dehydrogenase (ALDH) and finally to retinoic acid. After binding with cellular retinoic acid-binding protein (CRABP), RA is transferred into the nucleus and binds to retinoic acid receptors (RARs) and the retinoid X receptors (RXRs), causing conformational changes that activate these receptors. After activation, RAR/RXR heterodimers or RXR-RXR/RAR-RAR homodimers can be formed and bind to a promoter region known as the retinoic acid response element (RARE), resulting finally in an increase or decrease in the expression of specific genes. The catabolism of retinoids begins with cytochrome P450 cyp26-catalyzed hydroxylation of ATRA.

The target cells uptake RBP4-bound all-trans-retinol with the vitamin A receptor, stimulated by retinoic acid 6 (STRA6). Subsequently, retinol is bound to CRBP1 and undergoes oxidation in two steps, first to retinaldehyde and finally to all-trans-, 9-cis, and 13-cis retinoic acid. After binding with cellular retinoic acid binding protein (CRABP), RA is transferred into the nucleus and binds to the RAR or RXR, causing conformational changes that activate these receptors. After activation, RAR/RXR heterodimers or RXR-RXR/RAR-RAR homodimers can be formed and bind to a promoter region known as the retinoic acid response element (RARE). This starts a sequence that results in an increase or decrease in the expression of specific genes (Figure 2) [20,21,22,23,26,27].

## 5. RA and VSMCs

As we described above, under normal conditions, VSMCs exhibit a contractile phenotype that regulates normal vascular tone. In response to endothelial injury and vascular microenvironment changes, VSMCs display phenotypic plasticity, allowing them to differentiate into various cellular phenotypes, a state known as synthetic phenotypes. This phenotypic switching is well-known to play a central role in the process of atherosclerosis [5].

The retinoid receptors and the retinoid binding proteins are expressed in the heart both in the development of the embryo and in the adult [26]. A large number of in vivo and in vitro experiments have shown that RA has a pleiotropic effect that influences the migration, proliferation, and phenotypic switching of VSMCs.

Moreover, local and systemic delivery of RA can reduce the development of atherosclerotic plaques and effectively inhibit restenosis after vascular injury [17].

### 5.1. RA and VSMCs Proliferation

The proliferation of intimal VSMCs is essential for the development of neointimal mass and atherosclerotic plaque. Four decades ago, Peclo et al. applied RA at concentrations 10^−5^ to 10^−7^ M to cultured SMCs isolated from rat aortic tunica media, which enhanced the proliferation of VSMCs and increased the expression of L1 antigen, a specific surface antigen of SMCs [28]. Their results were not supported by later studies. Neuville et al. examined the effect of ATRA on inhibiting intimal formation. They showed that ATRA inhibits both medial and intimal thickening (IT) of SMC proliferation in vitro, with a more rapid effect on IT, likely due to its ability to metabolize retinoids induced by the presence of cellular retinol-binding protein-1 (CRBP-1). Furthermore, they demonstrated that ATRA and a RAR-a agonist resulted in a 76% reduction in IT and a 33% increase in lumen diameter without affecting medial SMCs after endothelial injury in rat carotid arteries [29]. Tran-Lundmark et al. showed that ATRA upregulates perlecan expression, a large heparan sulfate proteoglycan that acts as an important regulator for SMC growth, depending on the expression of its heparan-sulfate chain. More specifically, ATRA decreases the phosphorylation of phosphatase and tensin homolog (PTEN) and the levels of phosphorylated active protein kinase B (Akt) in wild-type cells, which promotes cell cycle arrest by antagonizing growth factor receptor- and integrin-stimulated signaling [30]. In addition, Streb et al. demonstrated that retinoids block the proliferation of VSMCs by the transcription of a specific A-kinase anchoring protein 12 (AKAP12) isoform (AKAP12β) in vascular SMC, a protein known for its tumor suppressive properties via PKA- mediated signaling [31]. Recently, it was found that direct activation of AMPK and inhibition of mTOR signaling by dose-dependent ATRA may inhibit neointimal hyperplasia and suppress VSMC proliferation and migration [32,33]. Moreover, a 72 h incubation of human SMC in 1 μΜ ATRA induced the expression of specific RA-target genes (RBP1, STRA6, CYP26B1, CRABP1, and RARβ), which inhibited cell proliferation. The activation of these genes participates in RA transport, uptake, conversion, and delivery to nuclear receptors, leading to the accumulation of RA derivatives [34].

Many studies have examined Kruppel-like factors (KLFs), a group of transcription factors that regulate cell growth, proliferation, and differentiation. KLF5 is a well-established pro-proliferative regulator, significantly expressed in adult VSMCs after vascular injury, enhancing the activation of various stimulator factors [35]. In in vitro SMC cultures, KLF5 was markedly increased in specimens with SMC outgrowth compared to the no outgrowth group (89 vs. 20%, *p* < 0.01), while earlier restenosis occurred in specimens with outgrowth [36]. Importantly, in KLF5(+/−) mice, less arterial wall stenosis was observed after vascular injury compared to wild types. They also demonstrated that KLF5 physically binds to RARa, promoting KLF5 transcriptional activity and neointimal formation [37]. Based on these results, Zhang et al. examined the effect of Am80, a synthetic RARa-specific agonist, both in vitro and in vivo. Co-immunoprecipitation analysis showed that Am80 successfully suppresses the interaction between KLF5 and RARa at cellular and tissue levels through KLF5 dephosphorylation in VSMCs, induced by the activation of PI3/Akt and p38 mitogen-activated protein kinase (MAPK) signaling pathways [38]. Additionally, Yu et al. examined the effect of oral administration of 10 mg/kg/day ATRA in a rabbit autogenous vein graft model after 2,4- and 8-week administration. ATRA treatment resulted in (1) a decrease in intima-media thickening of vein grafts and reduced expression of Kiel 67 (Ki67) protein, (2) inhibition of the growth activity of human umbilical vein smooth muscle cells (HUVSMCs) in a dose-dependent manner, probably mediated by retinoblastoma (RB) tumor suppressor protein E2F transcription factor (RB/E2F) (Rb-E2F) pathway, and (3) reduction in KLF5-RARa interaction and inhibition of the inducible NO synthase (iNO) expression, which is responsible for the activation of KLF5-downstream genes [39]. These findings indicate that KLF5-RARa interaction plays a crucial role in endothelial response to vascular injury, and RARa agonists may be used as potential drugs against neointima formation through KLF5 suppression. Moreover, KLF4 affects neointima formation by suppressing RARa expression and inhibiting PI3K/ERK signaling in cultured VSMC after synthetic Am80 administration [40].

Several studies have shown the effect of RA on various mitotic factors, inhibiting the proliferation of SMCs. Miano et al. demonstrated the expression of retinoid receptors in rat aortic SMC and the inhibition of SMC growth by the growth-inhibitory action of platelet-derived growth factor-BB by ATRA after 12 h of administration [41]. RA exhibits a stimulatory effect on elastin synthesis, with maximum stimulation of 2.8-fold at the concentration of 10^−6^ M for 24–48 h treatment and an increase in elastin mRNA levels, leading to the inhibition of cell proliferation [42]. Physiologically relevant concentrations of ATRA (IC_50_, 10 nM for serotonin, and IC_50_, 1 μM for serum) inhibited serotonin- and serum-induced VSMC proliferation in primary canine aortic VSMCs [43]. A 12 h exposure to ATRA reduced angiotensin I-receptor (AT_1_-R) gene expression in cultures VSMCs of rats’ thoracic aorta at both mRNA and protein levels. AT_1_-R was downregulated by ATRA through de novo protein synthesis dependent on the RAR/RXR heterodimer [44]. ATRA has the ability to suppress mitogenesis and extracellular signal-regulated protein kinase (ERK) activity in rat aortic cultured smooth cells mediated by endothelin stimulation, leading to a reduction in cell number in cultures, in contrast to the stimulatory effect induced when administered alone [45]. Finally, pretreatment with ATRA inhibited DNA synthesis mediated by basic fibroblast growth factor in a dose-dependent manner [46]. Although the mechanism of RA action on VSMC proliferation is not fully understood, there have been reports that RA acts by targeting cell cycle-related genes, such as cyclins and cyclin-dependent kinases (Cdks), which are essential for entering the cell cycle and for the subsequent progression to the G1 phase [46,47].

In summary, the above studies have clearly demonstrated an inhibitory effect on VSMC proliferation, either by affecting various mitogenic factors or by stimulating quiescent SMCs (Table 1).

### 5.2. RA and Migration

Migration of VSMCs is an essential step for neointima formation, primarily involving the degradation of extracellular matrix components regulated by matrix metalloproteinases (MMPs) or plasminogen activator (PA) systems [29].

RA downregulates the production of proMMP-1 in human aortic SMCs in a dose-independent manner through suppression of platelet-derived growth factor, suggesting that RA may contribute to the inhibition of VSMC migration [48]. Axel et al. examined the continuous and single-dose ATRA incubation in mono- and transfilter co-cultures of human arterial smooth muscle cells (haSMC). A counting assay after 7-day haSMC monoculture incubation resulted in an IC_50_ of 0.22 μΜ and an IC_max_ of 10.0 μM, with greater than 60% growth inhibition compared to control groups at a maximum concentration of 10.0 μM. In transfilter co-cultures induced by growth factors, non-stop administration of ATRA for 14 days resulted in reduced cell numbers on both filter sides with a greater effect on proliferation on the endothelial side at doses >/= 0.1 μΜ, while lower doses >/= 0.001 μM were effective for migration inhibition. Moreover, ATRA administration in monocultures showed a reduction in MMP-2 expression in unstimulated haSMC and in MMP-9 after >/=12 h of stimulation with phorbol ester. The researchers concluded on the migration and proliferation inhibitory effects of both non-stop and single-dose use of ATRA [16].

In another experimental model, oral administration of ATRA was examined in a mouse model of Kawasaki disease. Coronary stenosis and vasculitis were achieved by intraperitoneal injection of Lactobacillus casei cell wall extract into 5-week-old male C57BL/6 J mice. Two weeks later, oral ATRA at a dose of 30 mg/kg for 5 days per week for 14 weeks was administered, while the control group received corn oil alone. The elastin break score of external and internal elastin lumina was reduced, and MMP-9 protein was significantly suppressed in LCWE-induced mice. Coronary stenosis and inflammatory scores were also lower in the ATRA-treated group. Furthermore, human coronary artery smooth muscle cells were stimulated by platelet-derived growth factor subunit B homodimer (PDGF-BB), resulting in an augmentation of their area coverage by migration cells. Treatment with 0.1, 1.0, and 10 nM ATRA for 72 h showed a reduction in migration activity, with the most significant result of 49% in the group that received 10 nM ATRA. ELISA analysis showed significantly decreased total MMP-9 activity in the ATRA/PDGF-BB group, while no change was observed in the PDGF-BB group [49]. Similarly, Johst et al. observed a decrease in migrated cells in all haSMC groups incubated with RA compared to control groups, associated with reduced production of tenascin, as documented by immunostaining and downregulation of p44/p42 MAPKs. Tenascin is a matrix protein that promotes the transition from contractile VSMC to a motile phenotype, leading to a destabilization of cell-extracellular matrix interaction [50]. Although contradictory findings have been reported, indicating an increase in RA-induced migration [29], the above findings suggest that ATRA may play a role in the inhibition of VSMC migration (Table 2).

### 5.3. RA and Differentiation 

Phenotypic switching refers to the process whereby medial VSMC differentiate, proliferate, and migrate in response to various atherogenic stimuli. The transition of VSMC from the contractile type to the synthetic type contributes to the stability of atherogenic plaques by increasing the thickness of the protective fibrous cap [51]. Recently, Pan et al. demonstrated a transition of VSMC to an intermediate state known as SEM cells (stem cell, endothelial cell, monocyte) in an SMC-lineage tracing murine model, similar to those found in human atherosclerotic carotid arteries. SEM cells can differentiate into macrophage-like, fibroblast-like cells or revert to the initial SMC phenotype. The researchers administered ATRA, as an activator of RA signaling, to *ROSA26^ZsGreen1/+^*; *Ldlr^−/−^*; *Myh11-CreER^T2^* mice. They observed a ~40% reduction in the proportion of ZsGreen1^+^LY6A^+^LY6C1^+^ SEM cells among total ZsGreen1^+^ cells, while there was an increase in ZsGreen1^+^ cells in the protective fibrous cap. RNAscope analysis of vascular cell adhesion molecule-1 (*Vcam1*)-*Vcam1* in atherosclerotic BCA sections showed a ~70% decrease in *Vcam1*-stained SEM cells. These findings strongly suggest that ATRA treatment can attenuate SEM cell transition and suppress their dedifferentiation toward more differentiated SMC-derived cell types in atherosclerosis [52].

Under normal conditions, α-smooth muscle actin mRNA and protein synthesis present low concentrations in actively proliferating SMC [53]. ATRA has been shown to stimulate smooth muscle actin mRNA expression and myosin heavy chain (MHC) in haSMC, promoting a non-activated, fully differentiated phenotype that enhances the stabilization of atherosclerotic plaques [16,29].

Similarly, in vivo studies demonstrated an increase in smooth alpha-actin expression and myosin heavy chain following oral or local delivery of ATRA (Table 3), speculating the preservation of SMC ‘mature’ contractile phenotype [54,55,56]. 

Haller et al. proved an elevated expression of PKC and PKC-a in differentiated cultured SMC from rat aortas compared to dedifferentiated cells, which were characterized by α-actin and desmin expression in immunostaining and Western blot analysis. Subsequently, they incubated the cultures with retinoic acid and observed an increase in PKC-a expression and PKC activity, indicating an inhibitory action of RA on SMC dedifferentiation [57]. Another SMC differentiation marker is L-type Ca^2+^. In rat aortic (A7r5) SMC cultures, a decrease in the number of functional Ca^2+^ channels was observed in dedifferentiated cells, which was reversed after RA application [58]. Rogers et al. used human coronary artery SMC and showed that ATRA can inhibit the differentiation of SMC into an osteoblast-like phenotype through various mechanisms. Initially, they assessed changes in the osteogenic transcriptional program after ATRA application by measuring the mRNA levels of three transcriptional factors involved in the process of vascular calcification such as Runt-related transcription factor 2 (RUNX2), Msh homeobox 2 (MSX2), and SRY-box transcription factor 9 (SOX9) factors. Among the identified mechanisms are activation of RAR-a, regulation of tissue non-specific alkaline phosphatase, ATRA signaling gene expression, and an increase in G1a protein, all of which contribute to suppressing SMC calcification [59].

Another Kruppel-like factor, KLF4, has been shown to play a critical role in VSMC differentiation [40]. Wang et al. demonstrated that ATRA upregulates KLF4 and leads to an increase in the expression of differentiation marker genes (SM22a, alpha-SMA) while reducing the expression of dedifferentiation markers (SMemb) [60]. Various molecular mechanisms have been proposed for ATRA-mediated stimulation of KLF4 protein, including acetylation, phosphorylation [61,62], and transcriptional regulation of the Klf4 gene through functional interaction of RARa with KLF4, Sp1, and YB1 bound to GC boxes in the Klf4 promoter [61]. A novel factor similar to that of KLFs, ZFP580, was found to be induced by ATRA binding to RARa via the PI3K/Akt pathway. ZFP580 suppresses dedifferentiation, as evidenced by decreased expression of the dedifferentiation marker SMemb and the increased expression of SM22a and SMa-actin [51].

**Figure 3:**
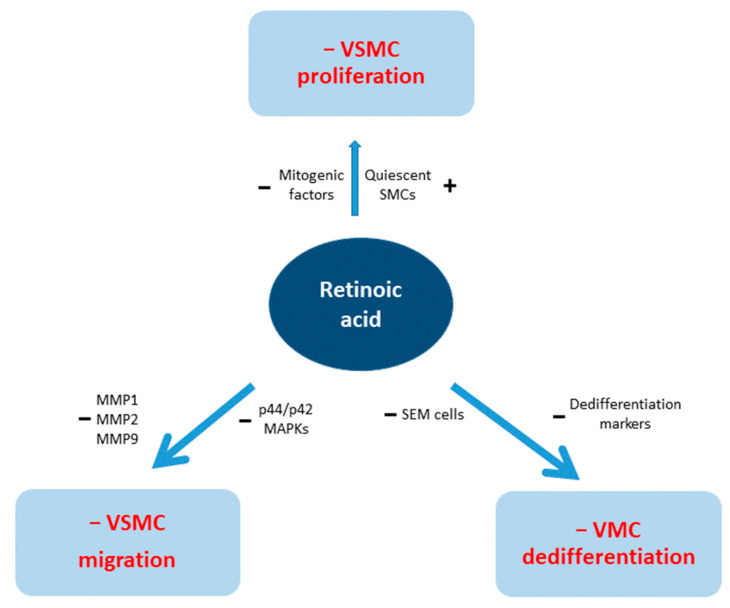
Action of retinoic acid on vascular smooth muscle cells (VSMCs). MMP = matrix metalloproteinase, MAPK = mitogen-activated protein kinase. SEM cells = stem cell, endothelial cell, monocyte.

The action of RA on VSMCs is summarized graphically in Figure 3. 

## 6. Future Directions and Conclusions

During the last decades, experimental in vitro and in vivo studies have shown that RA plays a role in both atherosclerotic processes and response to vascular injury. Experimental models have consistently demonstrated RA’s regulatory effect on VSMC proliferation, migration, and phenotypic switching, resulting in the inhibition and suppression of atherosclerotic plaque formation. It could be postulated that the next step in the research process could be human studies, especially in patients who underwent percutaneous coronary interventions with drug-eluting stents or drug-coated balloons. However, we still have a way to go before this target. The experimental models used in these studies (non-coronary arteries in rabbits and mice or rats) have some severe limitations [64]. It is known that the results of these models should be considered carefully, notably when the neointimal thickening was the “target” of the investigated therapy in arterial injury models [65]. On the other hand, porcine coronary arteries and their SMCs may be a more suitable experimental model regarding the properties of SMCs [66].

Moreover, the doses of RA used in experiments (usually ≥ 5 mg/kg/day) are impossible to be used in clinical cardiology since even much lower doses used in acute promyelocytic leukemia (proximate 1.5 mg/kg/d for 1^1/2^ to 3 months) are associated with common adverse effects and severe complications [11,67].

During the last two decades, local delivery of retinoids has been used through double-balloon catheters, porous polymeric membranes, vascular grafts, and stents [17,55,68,69].

However, the aforementioned limitations about the experimental models (and dose used) continued to exist.

It would be interesting to examine the local delivery of RA through a drug-eluting stent or balloon in a swine coronary artery experimental model. Till now, no retinoic drug-coated balloon has been tested in any experimental model. Moreover, devices for local delivery of retinoids must be compared with commercially used devices with paclitaxel and limus-family drugs (stents or balloons) regarding cytotoxicity and their effects on VSMC proliferation and regarding the time needed for dual antiplatelet therapy after the intervention.

In conclusion, RA signaling plays a role in the atherosclerotic progress. Various experimental models show a relatively common finding: the reduction in neointimal hyperplasia and stenosis or restenosis rate after injury and graft or stent placement in non-coronary arteries of rabbits, mice, or rats. These data indicate the need for further investigation of the usefulness of RA-related devices (stents or balloons) in the treatment of human coronary and peripheral lesions.

## Figures and Tables

**Table 1:** Experimental models of the effects of RA on the proliferation of VSMCs.

First Author (Year)	Experimental Model	Agent, Dose of Administration	Main Results	Other Results
Peclo M.M (1987) [28]	Cultured rat aortic SMC	RA 10^−5^–10^−7^ M	• Enhancement of proliferation of SMC • Delay of the exit of cells from the proliferative cycle • Higher saturation density of the culture	• Increased expression of L1
Neuville P. (1999) [29]	Cultured aortic media and intimal thickening rat SMC, angioplasty of rat carotid artery and thoracic aorta	10^−6^ final concentration, 0.5 mg/kg intraperitoneally/day for 14 days of RA	• Medial and intimal thickening inhibition • Proliferation inhibition by the presence of CRBP-1 • Increased migration and tissue-type plasminogen activator activity • Decreased α-smooth muscle actin in SMC cultured from the IT	• Reduced the intimal hyperplasia in the carotid artery in vivo
Tran-Lundmark K. (2015) [30]	Murine SMC cultures	2 μg/mL ATRA	• Regulation of SMC growth through upregulation of perlecan expression	• Activation and phosphorylation of PTEN and Akt in wild-type and mutant SMCs
Streb JW. (2011) [31]	Rat pulmonary artery SMC, rat aortic SMC, human coronary artery SMC, adult male mice model	In vitro: 2 × 10^−6^ of RA, 13-cis RA In vivo: 10 mg/kg ATRA	• Attenuation of SMC growth through inducible expression of AKAP12β via PKA-mediated signaling	
Zhang J. (2019) [32]	Common carotid artery ligation mouse model	10 mg/kg and 20 mg/kg of RA for 21 days	• Inhibition of neointima hyperplasia • Suppression of SMC proliferation and migration through direct activation of AMPK and inhibition of mTOR signaling	• Alteration of expression of proliferation-related in proteins (Cyclin D1, Cyclin D3, Cyclin A2, CDK4, CDK6)
Kim EJ. (2014) [33]	Human aortic SMC rat A7r5 Balloon injury rats	RORa, cholesterol sulfate	• Activation of AMPK-mTOR-S6K1 signaling pathway • Suppression of neointima formation after ballon injury	• Modulate the expression of cell-cycle-regulating factors and induces G1 arrest
Bilbija D. (2014) [34]	Human SMC	1 μM ATRA	• Inhibition of proliferation through expression of RA-target genes (RBP1, STRA6, CYP26B1, CRABP1 and RARβ)	
Zhang X. (2009) [38]	thoracic aorta VSMC rat model Ballon injury rats	2 μmol/LAm80 1 mg/kg/d	• Suppression of interaction between KLF5-RARa through dephosphorylation in VSMCs induced by the activation of PI3/Akt and p38 MAPK signaling pathways	• Induction of RARa expression • Inhibition of KLF5
Yu Y. (2021) [39]	rabbit autogenous vein graft model	10 mg/kg/day ATRA	• Decrease in intima-media thickening, • Reduced expression of Ki67 inhibition of the growth activity of HUVSMCs mediated by the Rb-E2F pathway	• Reduction in KLF5-RARa interaction • Inhibition of the iNO expression
Zheng B. (2009) [40]	Rat aortic VSMC	10 μΜ Am80, Am50	• Suppression of RARa expression and inhibition of PI3K/ERK signaling	• Klf4 inhibits the proliferation by PDGFRβ induced by PDGF-BB
Miano JM. (1996) [41]	Rat aortic SMC	2 × 10^−6^ mol/L	• inhibition of SMC growth by the growth-inhibitory action of PDGF-BB	• Expression of retinoid receptors in rat aortic SMC
Hayashi A. (1995) [42]	Chick embryos aortas SMC	10^−6^ RA	• Inhibition of cell proliferation through an increase in elastin mRNA levels	
Pakala R. (2000) [43]	Canine aortic SCM	RARγ-specific agonist	• Inhibition of serotonin- and serum-induced VSMC proliferation	• 1–10 nM ATRA inhibited serum- and 5-HT-induced ^3^[H]thymidine incorporation and cell number
Takeda K. (2000) [44]	Rat thoracic aorta SMC	1 mmol/L ATRA	• Downregulation of AT_1_-R mRNA through de novo protein synthesis-dependent on the RAR/RXR heterodimer	• Reduced AT_1_-R gene expression
Chen S. (1998) [45]	Rat aortic SMC	ARTA, RXR-, RAR-selective agonist	• Suppress mitogenesis and ERK activity mediated by endothelin stimulation	• Stimulates mitogenesis without changing ERK activity • Coincident activation of p21 expression
Kosaka C. (2001) [46]	Rat aortic SMC	1 mmol/l ATRA	• Inhibition of DNA synthesis mediated by basic fibroblast growth factor	• Suppression of the pRb kinase activities of Cdks
Wakino S. (2001) [47]	Human coronary SMC	TTNBP, ATRA, AGN4204, 9cRA	• Antiproliferative activity by regulation of G_1_/S cell cycle via inhibition of Rb phosphorylation	• Inhibition of DNA synthesis by stimulation of platelet-derived growth factor and insulin

9cRA: 9-cis stereoisomer, AKAP12β: A-Kinase Anchoring Protein 12, AMPK: adenosine monophosphate-dependent protein kinase, AT_1_-R: angiotensin 1 receptor, ATRA: all-trans retinoic acid, CDK4: cyclin-dependent kinase 4, CDK6: cyclin-dependent kinase 6, Cdks: cyclin-dependent Kinase, CRABP1: cellular retinoic acid binding protein 1, CRBP-1: cellular retinol-binding protein-1, CYP26B1: cytochrome P450, family 26, subfamily B, polypeptide 1, ERK: extracellular signal-regulated kinase, 5-HT: 5-hydroxytryptamine, HUVSMCs: human umbilical vein smooth muscle cells, iNO: inducible NO synthase, KLF5: krüppel-like factor 5, MAPK: mitogen-activated protein kinase, mRNA: messenger RNA, mTOR: mammalian target of rapamycin, PDGF-BB: platelet-derived growth factor-BB, PDGFRβ: platelet-derived growth factor receptor β, PI3K: phosphatidylinositol 3-kinase, PKA: protein kinase A, pRb: retinoblastoma protein, RBP1: retinol binding protein 1, RORa: retinoid-related orphan receptor A, RXR: retinoic X receptor, SMC: smooth muscle cell, STRA6: stimulated by retinoic acid gene 6, RA: retinoic acid, RARα: retinoic acid receptor α, RARβ: retinoic acid receptor β, RARγ: retinoic acid receptor γ.

**Table 2:** Experimental models of the effects of RA on the migration of VSMCs.

First Author (Year)	Experimental Model	Agent, Dose of Administration	Main Results	Other Results
Kato S. (1993) [48]	human aortic SMCs		• Downregulation of the production of proMMP-1 after treatment with PDGF	
Axel D. (2000) [16]	mono- and transfilter co-cultures of human arteries SMC	0.01 nM–10.0 mM atRA	• Migration and proliferation inhibition through reduction in MMP-2 and MMP-9 expression	• Decrease in mRNA expression of the glycoproteins thrombospondin-1, fibronectin
Suganuma E. (2021) [49]	Male mouse model of Kawasaki disease	30 mg/kg ATRA, oral administration	• Reduction in elastin break score of external and internal elastin lumina • Suppression of MMP-9 protein	• Lower coronary stenosis and inflammatory scores • Augmentation of their area coverage by migration cells after stimulation by platelet-derived PDGF-BB
Johst U. (2003) [50]	human aortic SMC	10^−6^ M, 10^−7^ M, and 10^−8^ M ATRA, 9cis RA	• Decrease in migrated cells associated with reduced production of tenascin and downregulation of p44/p42 MAPKs	• Increased G1-phase • Stronger expression of α-actin
Neuville P. (1999) [29]	Cultured aortic media and intimal thickening rat SMC, angioplasty of rat carotid artery and thoracic aorta	10^−6^ final concentration, 0.5 mg/kg intraperitoneally/day for 14 days of RA	• Increased migration and tissue-type plasminogen activator activity	• Reduced the intimal hyperplasia in the carotid artery in vivo

MAPKs: mitogen-activated protein kinases, MMP 2: matrix metalloproteinase-2, MMP 9: matrix metalloproteinase 9, PDGF: platelet-derived growth factor, PDGF-BB: platelet-derived growth factor-BB.

**Table 3:** Experimental models of the effects of RA on the dedifferentiation of VSMCs.

First Author (Year)	Experimental Model	Agent, Dose of Administration	Main Results	Other Results
Pan H. (2020) [52]	*ROSA26^ZsGreen1/+^*; *Ldlr^−/−^*; *Myh11-CreER^T2^* mice	10 μM ATRA for 72 h	• Attenuation of SEM cells transition • Suppression of SMC dedifferentiation • Reduction in atherosclerotic burden, • Promoted fibrous cap stability.	
Axel D. (2001) [16]	mono- and transfilter co-cultures of human arteries SMC	0.01 nM–10.0 mM ATRA	• Enhanced a-smooth muscle actin and heavy chain myosin expression	• Decrease in mRNA expression of the glycoproteins thrombospondin-1, fibronectin
Neuville P. (1999) [29]	Cultured aortic media and intimal thickening rat SMC, angioplasty of rat carotid artery and thoracic aorta	10^−6^ final concentration, 0.5 mg/kg intraperitoneally/day for 14 days of RA	• Decreased α-smooth muscle actin in SMC cultured from the IT	• Reduced the intimal hyperplasia in the carotid artery in vivo
Wiegman P.J. (2000) [54]	Balloon angioplasty on rabbits with focal femoral atherosclerosis	25 mg ATRA for the 3 days before and for 28 days after balloon injury	• More a-actin and desmin immunostaining	• Larger lumen area, internal elastic lamina, and external elastic lamina areas
Herdeg C. (2002) [55]	Right carotid artery of rabbits after induction of a fibromuscular plaque	10 mL, 10 μM ATRA, local delivery with double-balloon catheter	• More intense a-actin staining pattern	• Reduced early neointimal proliferation and extent of stenosis
Colbert M. (1996) [56]	Transgenic mouse carrying *lacZ* transgene		• Inducing and maintaining smooth muscle differentiation in the developing ductus arteriosus • Promote the expression of the adult vascular phenotype	• Appearance of smooth muscle myosin heavy chain isoform
Haller H. (1995) [57]	VSMC of rat aortas	10^−9^ mol/L ATRA for 12 and 48 h	• Inhibition of differentiation through increase in PKC-a expression and PKC activity	• Elevated expression of PKC and PKC-a in differentiated cultured SMC
Gollasch M. (1998) [58]	rat aortic (A7r5) VSMC cultures	10^−8^ M ATRA for 48 h	• Increase in the number of functional Ca^2+^ channel	• L-type Ca^2+^ channel is a novel marker for differentiation of VSMC • Correlation of L-type Ca^2+^ α1-subunits expression with alpha-SMA and SM-MHC expression
Rogers M. (2024) [59]	human coronary artery SMC	1 μmol/L ATRA, 1 μmol/L 9-cis RA	• Inhibition of SMC differentiation into osteoblast-like phenotype	
Wang C. (2008) [60]	VSMC of thoracic aorta of male rats	5, 10 or 20 mM of ATRA	• Inhibition of proliferation and migration of VSMCs through upregulation of KLF4, differentiation marker genes (SM22a, alpha-SMA) and KLF4 target genes p53	• Downregulation of SMemb
Meng F. (2009) [61]	VSMC of thoracic aorta of male rats	10 μM ATRA	• Induction of HDAC2 phosphorylation mediated by JNK signaling leading to the increase in KLF4 acetylation	• Inhibition of the interaction between KLF4 and HDAC2
Yu K. (2011) [62]	VSMC of thoracic aorta of male rats	5, 10, or 20 mM ATRA	• Activation of SM22α expression • Stimulation of KLF4 acetylation by induction of KLF4 phosphorylation	
Shi J. (2012) [63]	VSMC of thoracic aorta of male rats	10 μM ATRA	• RARα mediated ATRA-KLF4 expression	
Wei S. (2018) [51]	VSMC of thoracic aorta of rats	0, 5, 10, 20 μmol/L ATRA	• Suppression of dedifferentiation through RARα induced ZFP580 expression via the PI3K/Akt pathways	

alpha-SMA: alpha-smooth muscle actin, ATRA: all-trans retinoic acid, HDAC2: histone deacetylase 2, IT: intima thickening, KLF4: krüppel-like factor 4, PKC: protein kinase-C, SM22a: SMC: smooth muscle cell, SMemb: myosin heavy chain-B, SM-MHC: smooth muscle myosin heavy chain, PI3K: phosphatidylinositol 3-kinase, RARα: retinoic acid receptor α, VSMC: vascular smooth muscle cell.

## Abbreviations

ACVD = Atherosclerotic cardiovascular disease. AKAP12 = A-kinase anchoring protein 12. ALDH = Aldehyde dehydrogenase. Akt = protein kinase Β. AT1-R = Angiotensin I- receptor. ATRA = All-trans-retinoic acid. BCMO1 = b-carotene 15,15’-monooxygenase-1. Cdks = Cyclin-dependent kinases. CRABPs = Cellular retinoic acid-binding protein. CRBP = Cellular retinol-binding protein. Cyp26 = Cytochrome P450 26. ERK = Extracellular signal-regulated kinase. HASMC = Human aortic smooth muscle cell. HDAC = Histone deacetylase. IT = Intimal thickening. Ki67 = Kiel 67 protein. KLF5 = Krüeppel-like factor 5. LDLR = Low-density lipoprotein receptor. LRAT = Lecithin–retinol acyltransferase. MAPK = Mitogen-activated protein kinase. MMP = Matrix metalloproteinase. mRNA = Messenger ribonucleic acid. mTOR = Mammalian target of rapamycin. PDGF-BB = Platelet-derived growth factor subunit B. PI3K = Phosphatidylinositol kinase-3. PTEN = phosphatase and tensin homolog. RA = Retinoic acid. RAL = Retinaldehyde (retinal). RAR = Retinoic acid receptors. RARE = Retinoic acid response element. RB/E2F pathway = Retinoblastoma (RB) tumor suppressor protein E2F transcription factor pathway. RBP = Retinol-binding protein. RE = Retinyl esters. REH = Retinyl ester hydrolase. ROL = Retinol (vitamin A). RORa = Retinoid-related orphan receptor A. RXR = Retinoid X receptors. SMC = Smooth muscle cell. STRA6 = Vitamin A receptor stimulated by retinoic acid 6. TTR = Transthyretin. VCAM-1 = Vascular cell adhesion molecule. VSMCs = Vascular smooth muscle cells. ZFP = Zinc finger protein.

## References

[1] Geovanini G.R., Libby P. (2018). Atherosclerosis and Inflammation: Overview and Updates. Clin. Sci..

[2] Joseph P., Leong D., McKee M., Anand S.S., Schwalm J.-D., Teo K., Mente A., Yusuf S. (2017). Reducing the Global Burden of Cardiovascular Disease, Part 1: The Epidemiology and Risk Factors. Circ. Res..

[3] Byrne R.A., Rossello X., Coughlan J.J., Barbato E., Berry C., Chieffo A., Claeys M.J., Dan G.-A., Dweck M.R., Galbraith M. (2024). 2023 ESC Guidelines for the management of acute coronary syndromes. G. Ital. Cardiol..

[4] Hansson G.K., Hermansson A. (2011). The Immune System in Atherosclerosis. Nat. Immunol..

[5] Grootaert M.O.J., Bennett M.R. (2021). Vascular Smooth Muscle Cells in Atherosclerosis: Time for a Re-Assessment. Cardiovasc. Res..

[6] Chen R., McVey D.G., Shen D., Huang X., Ye S. (2023). Phenotypic Switching of Vascular Smooth Muscle Cells in Atherosclerosis. J. Am. Heart Assoc..

[7] Maguire E.M., Pearce S.W.A., Xiao Q. (2019). Foam Cell Formation: A New Target for Fighting Atherosclerosis and Cardiovascular Disease. Vasc. Pharmacol..

[8] Nakatani M., Takeyama Y., Shibata M., Yorozuya M., Suzuki H., Koba S., Katagiri T. (2003). Mechanisms of Restenosis after Coronary Intervention: Difference between Plain Old Balloon Angioplasty and Stenting. Cardiovasc. Pathol..

[9] Ghyselinck N.B., Duester G. (2019). Retinoic Acid Signaling Pathways. Development.

[10] Zawada D., Kornherr J., Meier A.B., Santamaria G., Dorn T., Nowak-Imialek M., Ortmann D., Zhang F., Lachmann M., Dreßen M. (2023). Retinoic Acid Signaling Modulation Guides in Vitro Specification of Human Heart Field-Specific Progenitor Pools. Nat. Commun..

[11] Hunsu V.O., Facey C.O.B., Fields J.Z., Boman B.M. (2021). Retinoids as Chemo-Preventive and Molecular-Targeted Anti-Cancer Therapies. Int. J. Mol. Sci..

[12] Oliveira L.d.M., Teixeira F.M.E., Sato M.N. (2018). Impact of Retinoic Acid on Immune Cells and Inflammatory Diseases. Mediat. Inflamm..

[13] Cassim Bawa F.N., Gopoju R., Xu Y., Hu S., Zhu Y., Chen S., Jadhav K., Zhang Y. (2022). Retinoic Acid Receptor Alpha (RARα) in Macrophages Protects from Diet-Induced Atherosclerosis in Mice. Cells.

[14] Maitra U., Parks J.S., Li L. (2009). An Innate Immunity Signaling Process Suppresses Macrophage ABCA1 Expression through IRAK-1-Mediated Downregulation of Retinoic Acid Receptor Alpha and NFATc2. Mol. Cell. Biol..

[15] Deng Q., Chen J. (2022). Potential Therapeutic Effect of All-Trans Retinoic Acid on Atherosclerosis. Biomolecules.

[16] Axel D.I., Frigge A., Dittmann J., Runge H., Spyridopoulos I., Riessen R., Viebahn R., Karsch K.R. (2001). All-Trans Retinoic Acid Regulates Proliferation, Migration, Differentiation, and Extracellular Matrix Turnover of Human Arterial Smooth Muscle Cells. Cardiovasc. Res..

[17] Samara I., Katsouras C.S., Semertzioglou A., Vratimos A., Moula A.I., Dimitriou C.A., Theofanis M., Papadimitropoulou T., Bouratzis V., Karanasiou G. (2022). Histopathological Evaluation of a Retinoic Acid Eluting Stent in a Rabbit Iliac Artery Model. Sci. Rep..

[18] IUPAC-IUB Joint Commission on Biochemical Nomenclature (JCBN) (1982). Nomenclature of Retinoids: Recommendations 1981. Eur. J. Biochem..

[19] Harrison E.H., Curley R.W. (2016). Carotenoids and Retinoids: Nomenclature, Chemistry, and Analysis. The Biochemistry of Retinoid Signaling II.

[20] Gudas L.J. (2022). Retinoid Metabolism: New Insights. J. Mol. Endocrinol..

[21] Brun P.-J., Yang K.J.Z., Lee S.-A., Yuen J.J., Blaner W.S. (2013). Retinoids: Potent Regulators of Metabolism. BioFactors.

[22] Lerner U.H. (2024). Vitamin A—Discovery, Metabolism, Receptor Signaling and Effects on Bone Mass and Fracture Susceptibility. Front. Endocrinol..

[23] Napoli J.L., Yoo H.S. (2020). Retinoid Metabolism and Functions Mediated by Retinoid Binding-Proteins. Methods Enzymol..

[24] Al Tanoury Z., Piskunov A., Rochette-Egly C. (2013). Vitamin A and Retinoid Signaling: Genomic and Nongenomic Effects. J. Lipid Res..

[25] le Maire A., Alvarez S., Shankaranarayanan P., de Lera A.R., Bourguet W., Gronemeyer H. (2012). Retinoid Receptors and Therapeutic Applications of RAR/RXR Modulators. Curr. Top. Med. Chem..

[26] Gudas L.J. (2022). Synthetic Retinoids Beyond Cancer Therapy. Annu. Rev. Pharmacol. Toxicol..

[27] Samara I., Moulas A.N., Karanasiou G., Papadimitropoulou T., Fotiadis D., Michalis L.K., Katsouras C.S. (2024). Is It Time for a Retinoic Acid-Eluting Stent or Retinoic Acid-Coated Balloon? Insights from Experimental Studies of Systemic and Local Delivery of Retinoids. Hell. J. Cardiol..

[28] Peclo M.M., Printseva O.Y. (1987). Retinoic Acid Enhances the Proliferation of Smooth Muscle Cells. Experientia.

[29] Neuville P., Yan Z., Gidlöf A., Pepper M.S., Hansson G.K., Gabbiani G., Sirsjö A. (1999). Retinoic Acid Regulates Arterial Smooth Muscle Cell Proliferation and Phenotypic Features in Vivo and in Vitro through an RARalpha-Dependent Signaling Pathway. Arterioscler. Thromb. Vasc. Biol..

[30] Tran-Lundmark K., Tannenberg P., Rauch B.H., Ekstrand J., Tran P.-K., Hedin U., Kinsella M.G. (2015). Perlecan Heparan Sulfate Is Required for the Inhibition of Smooth Muscle Cell Proliferation by All-Trans-Retinoic Acid. J. Cell. Physiol..

[31] Streb J.W., Long X., Lee T.-H., Sun Q., Kitchen C.M., Georger M.A., Slivano O.J., Blaner W.S., Carr D.W., Gelman I.H. (2011). Retinoid-Induced Expression and Activity of an Immediate Early Tumor Suppressor Gene in Vascular Smooth Muscle Cells. PLoS ONE.

[32] Zhang J., Deng B., Jiang X., Cai M., Liu N., Zhang S., Tan Y., Huang G., Jin W., Liu B. (2019). All-Trans-Retinoic Acid Suppresses Neointimal Hyperplasia and Inhibits Vascular Smooth Muscle Cell Proliferation and Migration via Activation of AMPK Signaling Pathway. Front. Pharmacol..

[33] Kim E.-J., Choi Y.-K., Han Y.-H., Kim H.-J., Lee I.-K., Lee M.-O. (2014). RORα Suppresses Proliferation of Vascular Smooth Muscle Cells through Activation of AMP-Activated Protein Kinase. Int. J. Cardiol..

[34] Bilbija D., Elmabsout A.A., Sagave J., Haugen F., Bastani N., Dahl C.P., Gullestad L., Sirsjö A., Blomhoff R., Valen G. (2014). Expression of Retinoic Acid Target Genes in Coronary Artery Disease. Int. J. Mol. Med..

[35] Aizawa K., Suzuki T., Kada N., Ishihara A., Kawai-Kowase K., Matsumura T., Sasaki K., Munemasa Y., Manabe I., Kurabayashi M. (2004). Regulation of Platelet-Derived Growth Factor-A Chain by Krüppel-like Factor 5: New Pathway of Cooperative Activation with Nuclear Factor-kappaB. J. Biol. Chem..

[36] Sakamoto H., Sakamaki T., Kanda T., Hoshino Y., Sawada Y., Sato M., Sato H., Oyama Y., Nakano A., Takase S. (2003). Smooth Muscle Cell Outgrowth from Coronary Atherectomy Specimens in Vitro Is Associated with Less Time to Restenosis and Expression of a Key Transcription Factor KLF5/BTEB2. Cardiology.

[37] Shindo T., Manabe I., Fukushima Y., Tobe K., Aizawa K., Miyamoto S., Kawai-Kowase K., Moriyama N., Imai Y., Kawakami H. (2002). Krüppel-like Zinc-Finger Transcription Factor KLF5/BTEB2 Is a Target for Angiotensin II Signaling and an Essential Regulator of Cardiovascular Remodeling. Nat. Med..

[38] Zhang X., Zheng B., Han M., Miao S., Wen J. (2009). Synthetic Retinoid Am80 Inhibits Interaction of KLF5 with RAR Alpha through Inducing KLF5 Dephosphorylation Mediated by the PI3K/Akt Signaling in Vascular Smooth Muscle Cells. FEBS Lett..

[39] Yu Y., Wang Y., Fei X., Song Z., Xie F., Yang F., Liu X., Xu Z., Wang G. (2021). All-Trans Retinoic Acid Prevented Vein Grafts Stenosis by Inhibiting Rb-E2F Mediated Cell Cycle Progression and KLF5-RARα Interaction in Human Vein Smooth Muscle Cells. Cardiovasc. Drugs Ther..

[40] Zheng B., Han M., Bernier M., Zhang X., Meng F., Miao S., He M., Zhao X., Wen J. (2009). Krüppel-like Factor 4 Inhibits Proliferation by Platelet-Derived Growth Factor Receptor Beta-Mediated, Not by Retinoic Acid Receptor Alpha-Mediated, Phosphatidylinositol 3-Kinase and ERK Signaling in Vascular Smooth Muscle Cells. J. Biol. Chem..

[41] Miano J.M., Topouzis S., Majesky M.W., Olson E.N. (1996). Retinoid Receptor Expression and All-Trans Retinoic Acid-Mediated Growth Inhibition in Vascular Smooth Muscle Cells. Circulation.

[42] Hayashi A., Suzuki T., Tajima S. (1995). Modulations of Elastin Expression and Cell Proliferation by Retinoids in Cultured Vascular Smooth Muscle Cells. J. Biochem..

[43] Pakala R., Benedict C.R. (2000). RAR Gamma Agonists Inhibit Proliferation of Vascular Smooth Muscle Cells. J. Cardiovasc. Pharmacol..

[44] Takeda K., Ichiki T., Funakoshi Y., Ito K., Takeshita A. (2000). Downregulation of Angiotensin II Type 1 Receptor by All-Trans Retinoic Acid in Vascular Smooth Muscle Cells. Hypertension.

[45] Chen S., Gardner D.G. (1998). Retinoic Acid Uses Divergent Mechanisms to Activate or Suppress Mitogenesis in Rat Aortic Smooth Muscle Cells. J. Clin. Investig..

[46] Kosaka C., Sasaguri T., Komiyama Y., Takahashi H. (2001). All-Trans Retinoic Acid Inhibits Vascular Smooth Muscle Cell Proliferation Targeting Multiple Genes for Cyclins and Cyclin-Dependent Kinases. Hypertens. Res..

[47] Wakino S., Kintscher U., Kim S., Jackson S., Yin F., Nagpal S., Chandraratna R.A., Hsueh W.A., Law R.E. (2001). Retinoids Inhibit Proliferation of Human Coronary Smooth Muscle Cells by Modulating Cell Cycle Regulators. Arterioscler. Thromb. Vasc. Biol..

[48] Kato S., Sasaguri Y., Morimatsu M. (1993). Down-Regulation in the Production of Matrix Metalloproteinase 1 by Human Aortic Intimal Smooth Muscle Cells. Biochem. Mol. Biol. Int..

[49] Suganuma E., Sato S., Honda S., Nakazawa A. (2021). All Trans Retinoic Acid Alleviates Coronary Stenosis by Regulating Smooth Muscle Cell Function in a Mouse Model of Kawasaki Disease. Sci. Rep..

[50] Johst U., Betsch A., Wiskirchen J., Schöber W., Vonthein R., Rinkert N., Kehlbach R., Claussen C.D., Duda S.H. (2003). All-Trans and 9-Cis Retinoid Acids Inhibit Proliferation, Migration, and Synthesis of Extracellular Matrix of Human Vascular Smooth Muscle Cells by Inducing Differentiation in Vitro. J. Cardiovasc. Pharmacol..

[51] Wei S., Zhang J., Han B., Liu J., Xiang X., Zhang M., Xia S., Zhang W., Zhang X. (2018). Novel Zinc Finger Transcription Factor ZFP580 Facilitates All-Trans Retinoic Acid -Induced Vascular Smooth Muscle Cells Differentiation by Rarα-Mediated PI3K/Akt and ERK Signaling. Cell. Physiol. Biochem..

[52] Pan H., Xue C., Auerbach B.J., Fan J., Bashore A.C., Cui J., Yang D.Y., Trignano S.B., Liu W., Shi J. (2020). Single-Cell Genomics Reveals a Novel Cell State During Smooth Muscle Cell Phenotypic Switching and Potential Therapeutic Targets for Atherosclerosis in Mouse and Human. Circulation.

[53] Patel S., Shi Y., Niculescu R., Chung E.H., Martin J.L., Zalewski A. (2000). Characteristics of Coronary Smooth Muscle Cells and Adventitial Fibroblasts. Circulation.

[54] Wiegman P.J., Barry W.L., McPherson J.A., McNamara C.A., Gimple L.W., Sanders J.M., Bishop G.G., Powers E.R., Ragosta M., Owens G.K. (2000). All-Trans-Retinoic Acid Limits Restenosis after Balloon Angioplasty in the Focally Atherosclerotic Rabbit: A Favorable Effect on Vessel Remodeling. Arterioscler. Thromb. Vasc. Biol..

[55] Herdeg C., Oberhoff M., Baumbach A., Schroeder S., Leitritz M., Blattner A., Siegel-Axel D.I., Meisner C., Karsch K.R. (2003). Effects of Local All-Trans-Retinoic Acid Delivery on Experimental Atherosclerosis in the Rabbit Carotid Artery. Cardiovasc. Res..

[56] Colbert M.C., Kirby M.L., Robbins J. (1996). Endogenous Retinoic Acid Signaling Colocalizes with Advanced Expression of the Adult Smooth Muscle Myosin Heavy Chain Isoform during Development of the Ductus Arteriosus. Circ. Res..

[57] Haller H., Lindschau C., Quass P., Distler A., Luft F.C. (1995). Differentiation of Vascular Smooth Muscle Cells and the Regulation of Protein Kinase C-Alpha. Circ. Res..

[58] Gollasch M., Haase H., Ried C., Lindschau C., Morano I., Luft F.C., Haller H. (1998). L-Type Calcium Channel Expression Depends on the Differentiated State of Vascular Smooth Muscle Cells. FASEB J..

[59] Rogers M.A., Chen J., Nallamshetty S., Pham T., Goto S., Muehlschlegel J.D., Libby P., Aikawa M., Aikawa E., Plutzky J. (2020). Retinoids Repress Human Cardiovascular Cell Calcification With Evidence for Distinct Selective Retinoid Modulator Effects. Arterioscler. Thromb. Vasc. Biol..

[60] Wang C., Han M., Zhao X.-M., Wen J.-K. (2008). Kruppel-like Factor 4 Is Required for the Expression of Vascular Smooth Muscle Cell Differentiation Marker Genes Induced by All-Trans Retinoic Acid. J. Biochem..

[61] Meng F., Han M., Zheng B., Wang C., Zhang R., Zhang X., Wen J. (2009). All-Trans Retinoic Acid Increases KLF4 Acetylation by Inducing HDAC2 Phosphorylation and Its Dissociation from KLF4 in Vascular Smooth Muscle Cells. Biochem. Biophys. Res. Commun..

[62] Yu K., Zheng B., Han M., Wen J. (2011). ATRA Activates and PDGF-BB Represses the SM22α Promoter through KLF4 Binding to, or Dissociating from, Its Cis-DNA Elements. Cardiovasc. Res..

[63] Shi J., Zheng B., Chen S., Ma G., Wen J. (2012). Retinoic Acid Receptor α Mediates All-Trans-Retinoic Acid-Induced Klf4 Gene Expression by Regulating Klf4 Promoter Activity in Vascular Smooth Muscle Cells. J. Biol. Chem..

[64] Schwartz S.M., O’Brien E.R., DeBlois D., Giachelli C.M. (1995). Relevance of Smooth Muscle Replication and Development to Vascular Disease. The Vascular Smooth Muscle Cell.

[65] Muller D.W.M., Ellis S.G., Topol E.J. (1992). Experimental Models of Coronary Artery Restenosis. J. Am. Coll. Cardiol..

[66] Christen T., Bochaton-Piallat M.-L., Neuville P., Rensen S., Redard M., Van Eys G., Gabbiani G. (1999). Cultured Porcine Coronary Artery Smooth Muscle Cells: A New Model With Advanced Differentiation. Circ. Res..

[67] Patatanian E., Thompson D.F. (2008). Retinoic Acid Syndrome: A Review. J. Clin. Pharm. Ther..

[68] Gregory E.K., Webb A.R., Vercammen J.M., Flynn M.E., Ameer G.A., Kibbe M.R. (2014). Periadventitial atRA Citrate-Based Polyester Membranes Reduce Neointimal Hyperplasia and Restenosis after Carotid Injury in Rats. Am. J. Physiol. Heart Circ. Physiol..

[69] Gregory E.K., Webb A., Vercammen J.M., Kelly M.E., Akar B., Van Lith R., Bahnson E.M., Jiang W., Ameer G.A., Kibbe M.R. (2018). Inhibiting Intimal Hyperplasia in Prosthetic Vascular Grafts via Immobilized All-Trans Retinoic Acid. J. Control. Release.

